# Cytotoxicity and Survival Fitness of Invasive *covS* Mutant of Group A *Streptococcus* in Phagocytic Cells

**DOI:** 10.3389/fmicb.2018.02592

**Published:** 2018-10-30

**Authors:** Chuan Chiang-Ni, Yong-An Shi, Chih-Ho Lai, Cheng-Hsun Chiu

**Affiliations:** ^1^Department of Microbiology and Immunology, College of Medicine, Chang Gung University, Taoyuan, Taiwan; ^2^Graduate Institute of Biomedical Sciences, College of Medicine, Chang Gung University, Taoyuan, Taiwan; ^3^Molecular Infectious Disease Research Center, Chang Gung Memorial Hospital, Taoyuan, Taiwan; ^4^Department of Pediatrics, Chang Gung Children’s Hospital, Taoyuan, Taiwan

**Keywords:** group A *Streptococcus*, CovR/CovS, phagocytic cell, clindamycin, intracellular survival

## Abstract

Group A streptococci (GAS) with spontaneous mutations in the CovR/CovS regulatory system are more invasive and related to severe manifestations. GAS can replicate inside phagocytic cells; therefore, phagocytic cells could serve as the niche to select invasive *covS* mutants. Nonetheless, the encapsulated *covS* mutant is resistant to phagocytosis. The fate of intracellular *covS* mutant in phagocytic cells and whether the intracellular *covS* mutant contributes to invasive infections are unclear. In this study, capsule-deficient (cap^-^) strains were utilized to study how intracellular bacteria interacted with phagocytic cells. Results from the competitive infection model showed that the cap^-^ *covS* mutant had better survival fitness than the cap^-^ wild-type strain in the PMA-activated U937 cells. In addition, the cap^-^ *covS* mutant caused more cell damages than the cap^-^ wild-type strain and encapsulated *covS* mutant. Furthermore, treatments with infected cells with clindamycin to inhibit the intracellular bacteria growth was more effective to reduce bacterial toxicity than utilized penicillin to kill the extracellular bacteria. These results not only suggest that the *covS* mutant could be selected from the intracellular niche of phagocytic cells but also indicating that inactivating or killing intracellular GAS may be critical to prevent invasive infection.

## Introduction

*Streptococcus pyogenes* (group A *Streptococcus*, GAS) is a Gram-positive human pathogen causing diseases including pharyngitis, scarlet fever, impetigo, cellulitis, necrotizing fasciitis, and toxic shock syndrome (Olsen and Musser, 2010). Severe manifestations such as necrotizing fasciitis and toxic shock syndrome are associated with high mortality and substantial economic burden (Olsen and Musser, 2010; Hughes et al., 2015; Stevens and Bryant, 2017). More importantly, an increasing number of scarlet fever and necrotizing fasciitis cases has been reported worldwide in recent decades (Athey et al., 2016; Engelthaler et al., 2016; Lee et al., 2017; Kim and Cheong, 2018).

Acquired prophages have been considered the critical genetic cause of increased GAS virulence and invasiveness (Beres et al., 2002; Aziz et al., 2004; Sumby et al., 2005). In another aspect, the growing evidence provided by next generation sequencing analyses suggests that spontaneous mutations in the regulatory proteins of GAS are highly related to the increase in bacterial invasiveness and severe invasive infections (Friaes et al., 2015b). For example, mutations that result in inactivating the CovR/CovS two-component regulatory system cause derepression of virulence factors such as hyaluronic acid capsule, streptolysin O (SLO), and DNase Sda1 (Sumby et al., 2006; Walker et al., 2007). Hyaluronic acid capsule is the key structure for GAS to resist phagocytosis (Stollerman and Dale, 2008). SLO is a pore-forming toxin that has important roles in GAS escaping from phagolysosome and resistance to intracellular killing mechanisms (O’Seaghdha and Wessels, 2013; O’Neill et al., 2016). Furthermore, DNases Sda1 degrades the DNA structure of neutrophil extracellular traps (NETs) to help GAS escape from NETs killing (Walker et al., 2007). Phagocytic cells are crucial to the early control of GAS infection (Goldmann et al., 2004). Therefore, *covS* and *covR* mutants are more resistance to phagocytic killing than the wild-type strain. In line with these studies, strains with spontaneous mutations in the *covR* or *covS* gene were more frequently isolated from patients with severe manifestations compared to patients with mild pharyngeal infections (Ikebe et al., 2010; Friaes et al., 2015b).

Neutrophil extracellular traps have been proposed as the critical pressure to select *covS* mutants during infection (Walker et al., 2007). Li et al. (2014) have shown that the depletion of neutrophils resulted in reduced frequency of isolated *covR* or *covS* mutants from the mouse infection model. GAS has ability to inhibit azurophilic granule fusion with phagosome and avoid ubiquitylation and recognition by the host autophagy pathway (Staali et al., 2006; Barnett et al., 2013). In line with these observations, several reports showed that GAS survive and even replicate in the intracellular niche of endothelial cells, human monocyte-derived macrophages, and polymorphonuclear leukocytes (Medina et al., 2003a; Staali et al., 2006; Hertzen et al., 2010; Lu et al., 2015; O’Neill et al., 2016). Spontaneous mutations occur while bacteria replicate, and mutant variants are selected by environmental stresses. Therefore, *covS* mutant might appear in the intracellular niche and be selected by the pressure of intracellular killing mechanisms. Nonetheless, the encapsulated *covS* mutant is resistant to phagocytosis (Sumby et al., 2006) and the fate of intracellular *covS* mutant in phagocytic cells is unknown.

The aim of this study was to elucidate whether inactivation of CovS affects the fitness of GAS in phagocytic cells and whether the intracellular *covS* mutant has roles in the pathogenesis of severe invasive GAS infections. Results from the competitive infection model showed that the *covS* mutant has better fitness than the wild-type strain in phagocytic cells. Furthermore, the intracellular *covS* mutant is more toxic to the phagocytic cells than the intracellular wild-type strain and encapsulated extracellular *covS* mutant. These results suggesting that the inactivation of CovR/CovS regulation plays prominently in interaction with phagocytic cells.

## Materials and Methods

### Bacterial Strains and Culture Cell Lines

GAS A20 (*emm1* type) and its animal-passage *covS* mutant AP3 strains were used in the previous study and shown in Tablle 1 (Chiang-Ni et al., 2016). Briefly, strain AP3 was isolated from the spleen of A20-infected BALB/c mouse (subcutaneous infection) after 3 days of infection. Strain AP3 was genome sequenced. In addition to the frameshift 143T deletion in the *covS* was identified, another five SNPs and an Indel were found in the repeat sequence regions of transposases or rRNA genes (data not shown). To be noted, *trans*-complementation of *covR/covS* in AP3 restored the expression of CovR-controlled genes to the levels similar to its parental A20 strain (Chiang-Ni et al., 2016, 2017). GAS strains were cultured in TSB supplemented with 5% yeast extract (Becton, Dickinson and Company, Sparks, MD, United States). *Escherichia coli* DH5α was purchased from Yeastern (Yeastern Biotech Co., Ltd., Taipei, Taiwan) and cultured in Luria-Bertani (LB) broth (Becton) at 37°C with vigorous aeration. When appropriate, the antibiotic chloramphenicol (25 and 3 μg/mL for *E. coli* and GAS, respectively) was used for selection. Human leukemic monocyte lymphoma cell line U937 was cultured in RPMI medium supplemented with 10% of fetal calf serum (FCS) (Invitrogen, Waltham, MA, United States) at 37°C with 5% CO_2_.

**Table 1:** Plasmids and bacterial strains used in this study.

Plasmid/Strain	Parental strain	Description	Reference
Vector 78	–	Chloramphenicol cassette	Chiang-Ni et al., 2016
pCN143	–	Temperature-sensitive vector	Chiang-Ni et al., 2016
pCN144	–	pCN143::SpeB_C192S_	This study
pCN158	–	pCN143 carried the marker-less *hasA* deletion DNA fragment	This study
pCN159	–	pCN143::*hasA*Δ*cm*	This study
pCN171	–	pCN143::*slo*Δ*cm*	This study
A20	—	*emm1*-type wild-type strain	Chiang-Ni et al., 2009
AP3	A20	Animal-passaged strain, *covS* mutant	Chiang-Ni et al., 2016
SCN156	A20	*hasA* mutant (Cm^S^)	This study
SCN157	AP3	*hasA* mutant (Cm^S^)	This study
SCN158	AP3	*hasA* mutant (Cm^R^)	This study
SCN162	A20	*hasA* mutant (Cm^R^)	This study
SCN176	SCN157	*slo* mutant	This study
SCN208	SCN162	SpeB_C192S_ mutant	This study


### Construction of *hasA*, *slo*, and SpeB_C192S_ Mutants

The *hasA* gene including its upstream (730 bp) and downstream (572 bp) regions was amplified using primers hasA-F-3 and hasA-R-3, and ligated into the temperature-sensitive vector pCN143 (Chiang-Ni et al., 2016) with the *Bam*HI site. The *hasA* gene was removed using inverted polymerase chain reaction (PCR) with primers del-hasA-F-SacII and del-hasA-R-SacII to generate pCN158. The chloramphenicol cassette of Vector 78 (Chiang-Ni et al., 2017) was amplified using primers Vec78_cat-F-SacII and Vec78_cat-R-SacII, and ligated into pCN158 with the *Sac*II site to generate pCN159. To construct the *slo* mutant, the *slo* gene including its upstream (872 bp) and downstream (936 bp) regions was amplified using primers Slo-F-3 and Slo-R-3, and ligated into pCN143 with the *Bam*HI site. The *slo* gene was removed using inverted PCR with primers del-slo-F-sacI and del-slo-R-sacI, and replaced by a chloramphenicol cassette from Vector 78 to generate pCN171. All primers used in this study are described in Tablle 2. Plasmids pCN158, pCN159, and pCN171 (Tablle 1) were transformed into GAS strains by electroporation, and transformants were selected using spectinomycin at 30°C. Transformants were incubated at 37°C with the spectinomycin selection to force plasmid integration via single homologous recombination. Finally, transformants were transferred in antibiotic-free plate at 30°C to force plasmid excision from the chromosome via a second recombination. Transformants with double homologous recombination were selected by changes in colony morphology or chloramphenicol resistance. The deletion of target gene in the transformants was confirmed by sequencing.

**Table 2:** Primers used in this study.

Primer	Sequence (5′ - 3′)#
hasA-F-3	cgggatccttccccattgcaagcatatc
hasA-R-3	cgggatcctgcaaatttttctgcgtctg
del-hasA-F-sacII	tccccgcggtaatatgtgcatcgagtagt
del-hasA-R-sacII	tccccgcggcacaattacacctctttctt
vec78_cat-F-sacII	tccccgcgggatagatttatgatatag
vec78_cat-R-sacII	tccccgcggatttattcagcaagtctt
Slo-F-3	gcgggatccgcggaaaatatagcgatgga
Slo-R-3	gcgggatcctgttaagaggttggggcaag
del-slo-F-sacI	tccgagctcggtttgaaccgcttggtaaa
del-slo-R-sacI	tccgagctctggagaagaagcagggaaaa
SpeB_C192S_-F-1	cgaagcttatgctggtaccgctgagatt
SpeB_C192S_-R-1	cgaagcttttacgtccgtcagcaccatc


*#Underline, restriction enzyme site.*

To construct SpeB_C192S_ mutation strain, the *speB* gene (640 bp) with the mutation in T574 was amplified using primers SpeB_C192S_-F-1 and SpeB_C192S_-R-1 (Tablle 2) from pET-21a::*speB*_T574A_ (Chen et al., 2003), and ligated into pCN143 with the *Hin*dIII site to construct pCN144 (Tablle 1). The plasmid pCN144 was transformed into SCN162 (*hasA* mutant of A20), and transformants were selected by spectinomycin (100 μg/mL) at 30°C. Transformants were incubated at 37°C to force plasmid integration via single homologous recombination. Finally, transformants in which the plasmid was excised from the chromosome via a second recombination were selected in antibiotic-free plate at 30°C. The protease-negative transformants were screened on skim-milk agar plate (Chiang-Ni et al., 2016). The SpeB_C192S_ mutation in the selected transformants was further verified by sequencing, and the strain was designated as SCN208 (Tablle 1).

### U937 Cells Infection Model

The U937 cell line is a suspension, oncogenic human monocyte cell line and has the potential of differentiating into either macrophages or dendritic cells (Lawrence and Natoli, 2011). PMA (phorbol 12-myristate 13-acetate) is a phorbol ester capable of transforming monocytic cells toward the macrophage pathway. After PMA treatments, U937 cells form tight clusters and attach to the plastic surface (Garcia et al., 1999). In the present study, U937 cells were treated FCS-containing RPMI medium with 15 nM of PMA (1.5 mM in DMSO; SI-D2650, Sigma-Aldrich, St. Louis, MO, United States) for 3 days to induce cell differentiation from monocyte-like to macrophage-like cells. Differentiated U937 macrophages were cultured in 6-well plates at a density of 1 × 10^6^ cell/mL. In experiments that were needed to inhibit phagocytosis, U937 macrophages were treated with 10 μg/mL cytochalasin D (10 mg/mL in DMSO; C8273, Sigma-Aldrich) for 1 h before infection, and the same concentration of cytochalasin D was present in the medium during the infection. The overnight bacterial cultures were transferred to fresh broth at 1:50 dilution to an optical density at 600 nm of 0.5–0.6. Bacteria were washed and resuspended in FCS-free RPMI medium and opsonized with human IgG (1 mg/mL; I8640, Sigma-Aldrich) at 37°C for 30 min before infection. PMA-activated U937 cells were infected with the opsonized GAS at multiplicity of infection (MOI) of 0.5, 5, or 50 in FCS-free RPMI medium with centrifugation at 335 ×*g* for 3 min to facilitate and synchronize the infection. After 30 min of infection, the infected U937 cells were washed and treated with gentamicin (100 μg/mL), penicillin (10 μg/mL), clindamycin (10 μg/mL), or penicillin plus clindamycin, depending on different experimental conditions. After infection, cells were washed with 1 × phosphate-buffered saline (PBS) twice and lysed with 0.01% Triton X-100 for 5 min. The cell lysates were serial diluted and plating on TSBY plate for determining the number of survival GAS.

### LDH Cytotoxicity Assay

Culture supernatants of infected cells were collected by centrifugation at 250 ×*g* for 5 min. The release of lactate dehydrogenase (LDH) was measured by CytoTox96^®^ Non-Radioactive Cytotoxicity Assay (Promega, Fitchburg, WI, United States), according to the manual. The cytotoxicity (%) was calculated as follows: [(sample LDH release-medium blank)/(maximum LDH release-medium blank)] × 100. The maximum LDH release was indicated as the LDH released from uninfected cells treated with lysis buffer for 45 min at 37°C.

### Hyaluronic Acid Capsule ELISA Assay

The hyaluronic acid capsule extraction was performed according to the method described previously (Henningham et al., 2014). Briefly, overnight culture bacteria were collected and washed with PBS. 400 μL of bacterial suspension was mixed with 1 mL of chloroform and shaken for 3 min in the Mini-BeadBeater (BioSpec Products Inc., Bartlesville, OK, United States). Supernatants were collected by centrifugation at 13,200 ×*g* for 20 min at 4°C and analyzed with the Hyaluronan DuoSet ELISA kit (DY3614-05, R&D Systems, Minneapolis, MN, United States) according to the manual.

### Western Blot

The bacterial culture was collected by centrifugation at 2,850 ×*g* for 10 min at 4°C and sterilized using a 0.22 μm filter. Bacterial culture supernatants (30 μL) were mixed with 6× protein loading dye and separated by 12% sodium dodecyl sulphate-polyacrylamide gel electrophoresis (SDS-PAGE). Proteins were transferred onto polyvinylidene difluoride (PVDF) membranes and the membranes were incubated with 5% skim milk in PBST buffer (1 × PBS containing 0.2% of Tween-20) at room temperature (25–30°C) for 1 h. SpeB and SLO were detected by anti-SpeB (Toxin Technology, Inc., Sarasota, FL, United States) and anti-SLO (Abcam, Cambridge, United Kingdom) antibodies, respectively. The blot was developed using Pierce ECL Western Blotting Substrate (Thermo Fisher Scientific Inc., Rockford, IL, United States), and the signal was detected using the Gel Doc XR+ system (BioRad, Hercules, CA, United States).

### Statistics Analysis

Statistical analysis was performed by using Prism software, version 5 (GraphPad, San Diego, CA, United States). Significant differences in multiple groups were determined using ANOVA. Post-test for ANOVA was analyzed by Tukey’s Honestly Significant Difference test. A *p*-value <0.05 was considered statistically significant.

## Results

### Inactivation of the Hyaluronic Acid Capsule Synthesis in the Animal-Passage *covS* Mutant Increases Its Interaction and Cytotoxicity With Phagocytic Cells at High MOI Conditions

**FIGURE 1:**
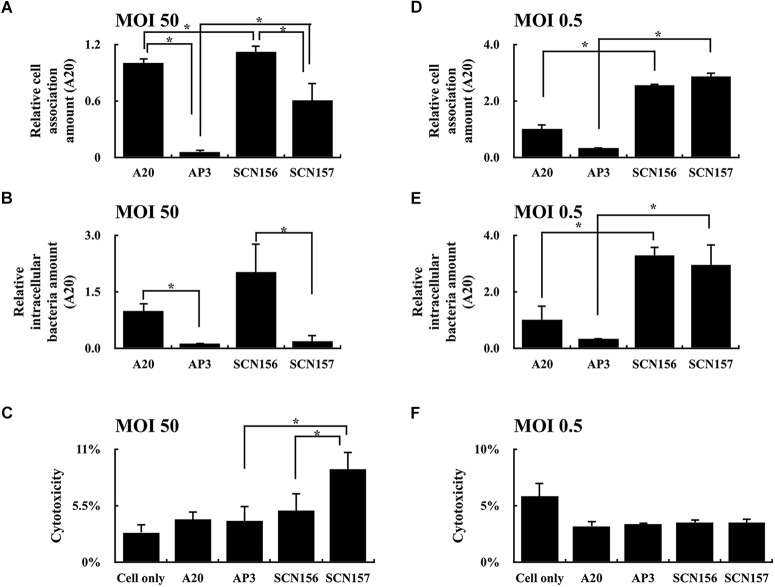
Cell-association and intracellular survival amount of wild-type A20 strain, animal-passage *covS* mutant AP3, and their capsule-deficient mutants (SCN156 and SCN157, respectively) in PMA-activated U937 cells. **(A,D)** Cell-association amount of A20, AP3, SCN156, and SCN157 with U937 cells. PMA-activated U937 cells were infected with the IgG-opsonized GAS strains at multiplicity of infection (MOI) of 50 or 0.5 for 30 min. The infected cells were washed after infection, and the number of cell-associated bacteria was determined using plating method. **(B,E)** Intracellular survival amount of A20, AP3, SCN156, and SCN157 with U937 cells. PMA-activated U937 cells were infected with the IgG-opsonized GAS strains at MOI of 50 or 0.5 for 30 min. The infected cells were washed after infection and incubated in gentamicin-containing medium for 45 min. After gentamicin treatment, the infected cells were washed, and the number of intracellular bacteria was determined using plating method. **(C,F)** Cytotoxicity of A20, AP3, SCN156, and SCN157 to U937 cells. PMA-activated U937 cells were infected with the IgG-opsonized bacteria at MOI of 50 or 0.5 for 45 min, and culture supernatants were collected for evaluating the cytotoxicity by lactic dehydrogenase (LDH) assay. Error bars represent the standard deviations. ^∗^*p* < 0.05.

The increased production of hyaluronic acid capsule in the *covS* mutant contributes to enhancing bacterial resistance to phagocytosis (Sumby et al., 2006). In order to evaluate how the animal-passage *covS* mutant (AP3) interacts with phagocytic cells, *hasA* mutants of wild-type A20 strain and AP3 were constructed. The concentration of hyaluronic acid capsule in the extract from A20 and AP3 was 0.96 ± 0.14 and 215.29 ± 57.36 ng/mL, respectively. In addition, the hyaluronic acid capsule was not detected (under the detection limits) in extracts from A20 and AP3 *hasA* mutants. PMA-activated U937 cells were infected with the IgG-opsonized A20, AP3, and their *hasA* mutants at MOI of 50 at 37°C for 30 min, and the number of cell-associated bacteria was analyzed using plating method. As expected, the number of cell-associated *hasA* mutant of AP3 (SCN157) was increased significantly compared to that of AP3 (Figure 1A), indicating that inactivation of hyaluronic acid capsule production increases bacteria binding to or being phagocytosed by phagocytic cells. Nonetheless, although the number of cell-associated SCN157 and SCN156 (*hasA* mutant of A20) was comparable, fewer viable intracellular SCN157 could be recovered after another 45 min of incubation (Figure 1B). In addition, we found that SCN157 caused more cell damage compared to other strains under microscope observations (Supplementary Figure 1), suggesting that SCN157 is toxic to PMA-activated U937 cells and therefore fewer intracellular SCN157 could be recovered after infection. The cytotoxicity of A20, AP3, SCN156, and SCN157 on PMA-activated U937 cells was further evaluated using the LDH assay. PMA-activated U937 cells were infected with these strains at MOI = 50 for 45 min, and supernatants were collected for this assay. Results showed that the cell damage in SCN157-infected cells was 2.46- and 1.8-fold higher as compared to that in cells infected with its parental AP3 strain and SCN156, respectively (Figure 1C). To evaluate whether the decrease in the number of intracellular SCN157 was related to its cytotoxicity, the cell-association and intracellular survival amount and cytotoxicity of these GAS strains under low MOI conditions (MOI = 0.5) were further evaluated. Results showed that SCN156 and SCN157 had similar cytotoxicity, cell-association, and intracellular survival amounts in PMA-activated U937 cells (Figures 1D–F). These results indicate that deletion of *hasA* increases the interaction between bacteria and phagocytic cells. In addition, the decrease in the number of intracellular SCN157 at MOI = 50 can be related to its increased cytotoxicity (Figures 1B,C).

### The Survival Fitness of Capsule-Deficient A20 and AP3 Strains in Culture Broth and PMA-Activated U937 Cells

**FIGURE 2:**
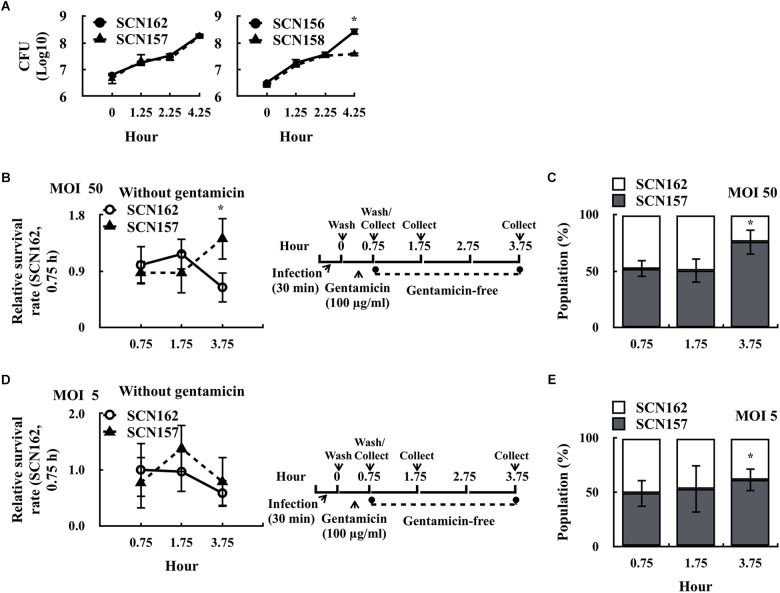
Survival fitness of the capsule-deficient A20 and AP3 in the culture broth and PMA-activated U937 cells. **(A)** Survival fitness of the capsule-deficient A20 (SCN156 [Cm^S^] and SCN162 [Cm^R^]) and AP3 (SCN157 [Cm^S^] and SCN158 [Cm^R^]) in trypticase soy broth with yeast extract (TSBY). An equal number of A20- and AP3-derivative strains was mixed and inoculated in TSBY. After 1.25–4.25 h of incubation, the number of A20 and AP3 mutants was determined using plating method. **(B,D)** Survival fitness of the capsule-deficient A20 (SCN162 [Cm^R^]) and AP3 (SCN157 [Cm^S^]) in PMA-activated U937 cells at multiplicity of infection (MOI) of 50 and 5 conditions. PMA-activated U937 cells were infected with mixed SCN162 and SCN157. The infection protocol is shown in the right panel. **(C,E)** The proportion of SCN157 and SCN162 in the recovered bacterial population after cells were infected with mixed SCN162 and SCN157 at MOI of 50 and 5 conditions. Cm: chloramphenicol. Error bars represent the standard deviations. ^∗^*p* < 0.05.

Gentamicin cannot penetrate through the mammalian cell membrane (Elsinghorst, 1994; Barnett et al., 2013). If the cell membrane is damaged, intracellular bacteria could be exposed to gentamicin and complicates the ability to evaluate bacterial survival fitness in phagocytic cells. We found that the fewer intracellular viable SCN157 was observed under high MOI (MOI = 50) but not low MOI (MOI = 0.5) conditions, suggesting that the increase in cytotoxicity of SCN157 could be related to the increase in susceptibility of intracellular SCN157 to gentamicin treatment (Supplementary Figure 2). In addition, this property may provide SCN157 with better resistance to phagocytic killing. SCN156 and SCN157 are marker-less *hasA* mutants. In order to compare the survival fitness of the capsule-deficient wild-type and *covS* mutant strains by competitive infection model, a chloramphenicol-resistant (Cm^R^) *hasA* mutant of A20 (SCN162) and AP3 (SCN158) was constructed. The growth activity of selected strains in culture broth was first compared. An equal number of SCN162 (Cm^R^) and SCN157 [Cm-susceptible (Cm^S^)] or SCN156 (Cm^S^) and SCN158 (Cm^R^) was mixed and inoculated in trypticase soy broth with yeast extract (TSBY). After incubation, the number of capsule-deficient A20 and AP3 was determined by plating bacteria on TSBY plates with or without Cm selection. Results showed that the growth activity of SCN162 and SCN157 was similar under broth culture conditions (Figure 2A). Nonetheless, SCN158 showed a decrease in the growth activity compared to SCN156 after 2.25–4.25 h of incubation (Figure 2A).

Based on these results, SCN162 and SCN157 were utilized for the following analyses. PMA-activated U937 cells were infected with mixed SCN162 and SCN157 (IgG-opsonized) at MOI = 50 for 30 min, and the number of survived bacteria was evaluated. At MOI = 50, we found that the survival rate of SCN157, but not SCN162, was increased after 3.75 h of incubation (Figure 2B). Furthermore, the changes in the proportion of SCN162 and SCN157 in the recovered bacterial population were analyzed after 0.75–3.75 h of incubation. After 0.75 and 1.75 h of incubation, a similar proportion of SCN162 and SCN157 was found in the recovered bacterial population (Figure 2C). Nonetheless, after 3.75 h of incubation, the proportion of SCN157 increased to 75% in the recovered bacterial population (Figure 2C). Under MOI = 0.5 conditions, both SCN156 and SCN157 does not cause damages to PMA-activated cells (Supplementary Figure 2). With the infection protocol described in Figure 2D, SCN162 and SCN157 caused the additional 1.6% ± 0.45 and 0.95% ± 0.17% of cell damages compared to the uninfected group after 3.75 h of incubation at the MOI = 5 conditions, respectively. Therefore, MOI = 5 was selected as the low MOI conditions for this competitive infection model. At MOI = 5, the survival rate of SCN162 and SCN157 was similar after 0.75–3.75 h of incubation (Figure 2D); while, the proportion of SCN157 in the recovered bacteria population increased from 49.12 to 61.67% after 0.75–3.75 h of incubation (Figure 2E). These results suggest that SCN157 has a better survival fitness than that of SCN162 in PMA-activated U937 cells.

### Effects of Cytochalasin D Treatment and Inactivation of SpeB and SLO on Cytotoxicity in PMA-Activated U937 Cells

**FIGURE 3:**
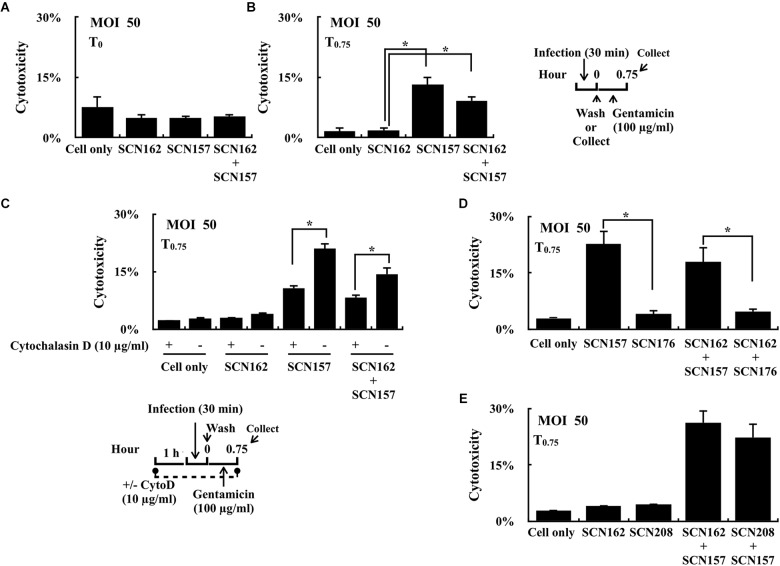
Cytotoxicity of the capsule-deficient A20 and AP3 (SCN162 and SCN157, respectively), SpeB_C192S_ mutant of SCN162 (SCN208), and *slo* mutant of SCN157 (SCN176) on PMA-activated U937 cells with or without cytochalasin D during infection. **(A,B)** Cytotoxicity of SCN162 and SCN157 on PMA-activated U937 cells after 0.5 h (T_0_) and 0.75 h (T_0.75_) of infection. The right panel shows the infection protocol of **(A,B,D,E)**. **(C)** Cytotoxicity of SCN162 and SCN157 on PMA-activated U937 cells with or without cytochalasin D (CytoD) treatment. The lower panel shows the infection protocol. **(D,E)** Cytotoxicity of SCN162, SCN157, SCN176, and SCN208 on PMA-activated U937 cells. Error bars represent the standard deviations. ^∗^*p* < 0.05.

Strain SCN157 is more cytotoxic to PMA-activated U937 cells compared to SCN156 after 1.75 h of infection at the MOI = 50 conditions (Supplementary Figure 2); however, increase in cell damage was not observed in cells infected with AP3 strain for 45 min (compared to A20; Figure 1C) and SCN157 for 30 min (compared to SCN162; Figure 3A). However, after additional 45 min of gentamicin treatment, an increased damage in cells infected with SCN157 and mixed SCN162 and SCN157 was observed (Figure 3B). These results suggest that intracellular SCN157 is more cytotoxic than intracellular SCN162 and extracellular bacteria. Therefore, cytochalasin D was utilized to inhibit phagocytosis, and the cytotoxicity of extracellular and intracellular bacteria in PMA-activated U937 cells was compared. Results showed that cytochalasin D treatment did not influence the cytotoxicity of SCN162 on PMA-activated U937 cells (Figure 3C). Nonetheless, cytochalasin D-treated cells showed 1.94- and 1.7-fold decrease in cell damage compared to untreated cells after SCN157 or mixed SCN162 and SCN157 infection, respectively (Figure 3C). These results not only support that extracellular *covS* mutant is cytotoxic (Hancz et al., 2017), but also suggest that intracellular *covS* mutant contributes significantly to causing cell damages.

Mutation in the *covS* gene leads to the repression of SpeB but upregulation of SLO expression (Sumby et al., 2006). Both SpeB and SLO are involved in bacterial resistance to intracellular killing (Barnett et al., 2013; O’Seaghdha and Wessels, 2013; Lu et al., 2015; O’Neill et al., 2016). Therefore, the effects of the repression of SpeB in SCN162 and upregulation of SLO in SCN157 on contributing to bacterial cytotoxicity in PMA-activated U937 cells was further elucidated. PMA-activated U937 cells were similarly infected with the *slo* mutant of SCN157 (SCN176) and SpeB protease-inactivated mutant of SCN162 (SpeB_C192S_, SCN208), and the cytotoxicity of these bacterial strains on U937 cells was evaluated. To be noted, the inactivation of SLO expression and SpeB protease activity in SCN176 and SCN208 were confirmed by western blotting (Supplementary Figure 3). Results showed that the cytotoxicity of SCN176 to PMA-activated U937 cells was decreased by 5.52-fold compared to that of its parental SCN157 strain (Figure 3D). In addition, the 3.81-fold decrease in cell damage was observed in cells infected with mixed SCN162 and SCN176 compared to cells infected with mixed SCN162 and SCN157 (Figure 3D). However, there was no significant difference in the cytotoxicity of SCN208 and its parental SCN162 strain (Figure 3E). Furthermore, the damage of cells infected with mixed SCN157 and SCN162 and mixed SCN157 and SCN208 was similar (Figure 3E). These results suggest that SLO but not SpeB is critical for the bacterial cytotoxicity on PMA-activated U937 cells.

### SLO Contributes to the Increase of Intracellular Survival of the Capsule-Deficient AP3 Strain

**FIGURE 4:**
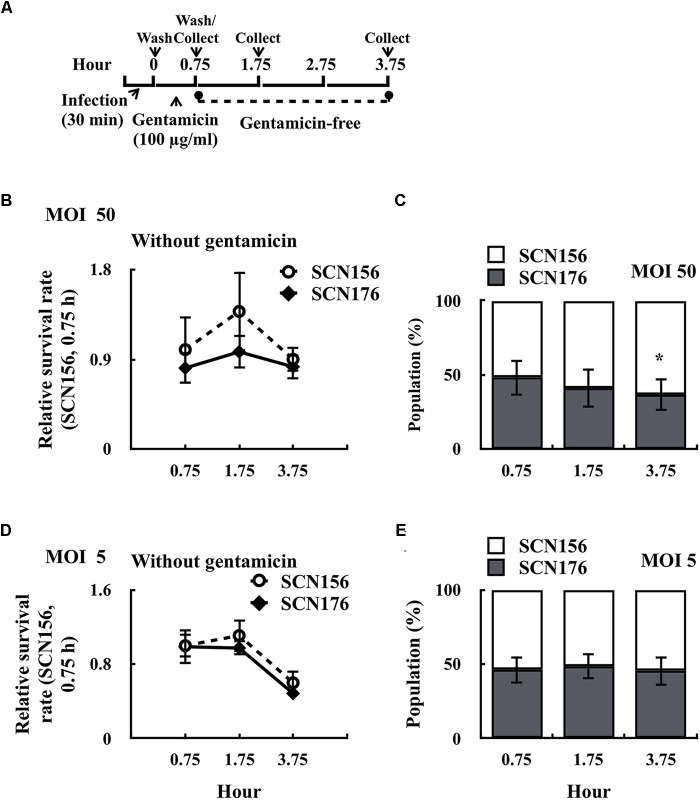
Survival fitness of the capsule-deficient A20 (SCN156), AP3 (SCN157), and *slo* mutants of SCN157 (SCN176) on PMA-activated U937 cells. PMA-activated U937 cells were infected with mixed (1:1 ratio) SCN156 and SCN176 at multiplicity of infection (MOI) of 50 or 5 as shown in panel **(A)**. **(B,D)** Survival fitness of SCN156 (Cm^R^) and SCN176 (Cm^S^) at MOI of 50 or 5 in gentamicin-free incubation conditions. **(C,E)** The proportion of SCN156 and SCN176 in the recovered bacterial population at MOI of 50 or 5 in gentamicin-free incubation conditions. Error bars represent the standard deviations. ^∗^*p* < 0.05.

Results shown in Figure 2 indicated that the capsule-deficient AP3 (SCN157) had better fitness in phagocytic cells than the capsule-deficient A20 strain. Furthermore, the *slo* mutant of SCN157 showed a significant decrease in cytotoxicity compared to its parental strain (Figure 3D). These results suggest that SLO is a critical factor for the increase in survival fitness of SCN157 in this competitive infection model. Therefore, the number of survived SCN156 and *slo* mutant of SCN157 (SCN176) with PMA-activated U937 cells was further elucidated (Figure 4A). Results showed that the survival rate of SCN156 and SCN176 was similar after 0.75–3.75 h of infection at both MOI = 50 and MOI = 5 (Figures 4B,D). In addition, at MOI = 50, the proportion of SCN176 in the recovered bacterial population decreased gradually from 48.2 to 36.3% after 0.75–3.75 h of infection (Figure 4C). At MOI = 5, the survival rate of SCN156 and SCN176 decreased after 3.75 h of infection, and the proportion of these strains in the bacterial population was similar after 0.75–3.75 h of infection (Figures 4D,E). These results indicated that inactivation of SLO expression in *covS* mutant decreased its activity to compete with SCN156 in PMA-activated U937 cells.

### Clindamycin but Not Penicillin Treatment Effectively Inhibits the Cytotoxicity of the Intracellular Capsule-Deficient AP3 Strain

**FIGURE 5:**
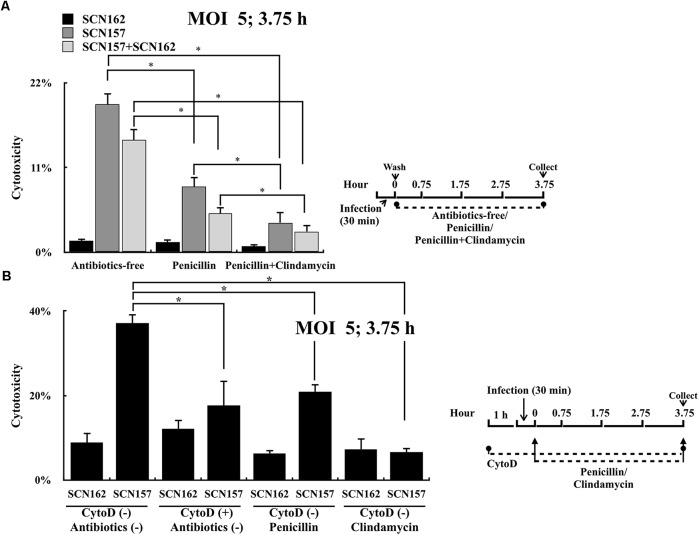
Cytotoxicity of the capsule-deficient A20 (SCN162) and AP3 (SCN157) on PMA-activated U937 cells with or without penicillin, clindamycin, and cytochalasin D treatment after infection. **(A)** Cytotoxicity of SCN162 and SCN157 on PMA-activated U937 cells with or without antibiotic treatment after 3.75 h of infection. **(B)** Cytotoxicity of SCN162 and SCN157 on PMA-activated U937 cells with or without cytochalasin D or antibiotic treatments after 3.75 h of infection. The right panels of **(A,B)** show the infection protocols. Error bars represent the standard deviations. ^∗^*p* < 0.05.

GAS is susceptible to penicillin; however, clindamycin is strongly recommended for treating patients with severe manifestations such as necrotizing fasciitis (Stevens et al., 2014). Unlike penicillin that is unable to penetrate through the mammalian cell membrane efficiently (Renard et al., 1987), clindamycin can reach high intracellular levels in phagocytic cells. Results shown in Figure 3C suggest that intracellular, but not extracellular capsule-deficient *covS* mutant is critical for causing cell damages. We proposed that clindamycin could be more effective in inhibiting bacterial cytotoxicity than penicillin. To prove this, PMA-activated U937 cells were infected with SCN162 and SCN157 at MOI = 50, and the infected cells were treated with penicillin or clindamycin. However, penicillin (10 μg/mL) and clindamycin (10–100 μg/mL) treatments did not reduce the cell damage in SCN162- and SCN157-infected cells (data not shown). This high MOI conditions could result in the extensive cell damage that cannot be rescued by the antibiotic treatments. Therefore, the effects of penicillin and clindamycin treatments on reducing bacterial cytotoxicity were evaluated at MOI = 5. Results showed that SCN162 had low cytotoxicity, and the supplementation of antibiotics after infection showed no effects on reducing SCN162-mediated cell damage (Figure 5A). Treatment of SCN157-infected cells with penicillin and penicillin plus clindamycin showed 2.25- and 5-fold decrease in cell damage, respectively (Figure 5A). Furthermore, similar results were also observed in PMA-activated U937 cells infected with mixed SCN162 and SCN157 (Figure 5A).

In order to elucidate whether clindamycin could reduce the cytotoxicity caused by intracellular bacteria, SCN162- and SCN157-mediated cell damage with or without cytochalasin D was compared. Similar to the results shown in Figures 3C, 5A, cytochalasin D or antibiotic treatments did not reduce SCN162-mediated cell damage (Figure 5B). Cytochalasin D and penicillin treatment reduced the SCN157-mediated cell damage by approximately 2-fold compared to the untreated conditions. Furthermore, clindamycin treatment reduced the SCN157-mediated cell damage by approximately 5.5-fold compared to the untreated conditions (Figure 5B). These results support that the intracellular SCN157 plays important roles in causing cell damage. More importantly, virulence of intracellular SCN157 could be inhibited effectively by clindamycin, attenuating bacterial cytotoxicity.

## Discussion

Mutations in the *covR* and *covS* gene in GAS result in increase in bacterial virulence and are associated with severe manifestations (Sumby et al., 2006; Ikebe et al., 2010). Although the immune system has been considered an important pressure to select *covR* and *covS* mutants during infection (Li et al., 2014), the specific environmental niche or stress required for selecting these mutants has not been comprehensively investigated. Results from the present study showed that the intracellular animal-passage *covS* mutant has better survival fitness than the wild-type strain in phagocytic cells. In addition, the intracellular *covS* mutant has more important roles in causing cell damages than the intracellular wild-type strain and extracellular *covS* mutant. These findings not only suggest that *covS* mutants can be selected in the intracellular niche of phagocytic cells, but also indicate that intracellular GAS can have important roles in invasive infections.

Mutations in the *covR* or *covS* gene result in the overproduction of hyaluronic acid capsule, and these mutants are highly resistant to phagocytosis (Ravins et al., 2000; Sumby et al., 2006). Nonetheless, pathogen variants rise while bacteria replicate. GAS has been shown to survive and replicate in the intracellular niche of different cell types (Medina et al., 2003b; Barnett et al., 2013; Lu et al., 2015). The hyaluronic acid capsule has no critical roles in GAS intracellular survival (Hertzen et al., 2010). Furthermore, capsule-deficient *emm4*- and *emm89*-type strains were isolated from patients with invasive GAS infections (Henningham et al., 2014; Friaes et al., 2015a; Zhu et al., 2015), suggesting that the hyaluronic acid capsule is dispensable for GAS to cause severe manifestations. Although we artificially attenuated the ability of GAS to resist phagocytosis by inactivating the capsule production, these mutants still provided critical information about how intracellular GAS interacted with phagocytic cells. Results from this study showed that the intracellular capsule-deficient animal-passage *covS* mutant caused more cell damage than the intracellular capsule-deficient wild-type and extracellular animal-passage *covS* mutant (Figures 1C, 3C). In addition, inactivation of the SLO expression in the capsule-deficient animal-passage *covS* mutant reduced its cytotoxicity (Figure 3D). These findings are consistent with the previously reported model, which is the cytotoxic effect of SLO depends on phagocytic uptake of GAS (Love et al., 2012). In the present study, our competitive infection model further showed that the capsule-deficient wild-type strain was outcompeted by the capsule-deficient animal-passage *covS* mutant in phagocytic cells (Figures 2B,C), and SLO is the determinant factor to provide selective advantages for intracellular GAS during infection (Figure 4). Therefore, these results not only suggest that the increased SLO production of the *covS* mutant is critical to enhance its survival fitness in phagocytic cells but indicating that intracellular *covS* mutant may contribute significantly to the increase in bacterial invasiveness during infection.

Mutations in the *covS* gene result in the upregulation of SLO, but repress SpeB production (Sumby et al., 2006). SpeB is an important virulence factor that contributes to severe tissue damage and invasive infection (Lukomski et al., 1997; Olsen and Musser, 2010; Olsen et al., 2015). In addition, the SpeB protease was reported to help M1T1 clone of GAS escape from intracellular autophagosome killing (Barnett et al., 2013). Although *covS* mutant is considered an invasive strain, whether the inactivation of SpeB in this mutant contributes directly to increased virulence and invasiveness is still under debate (Olsen et al., 2015). The *emm1*-type A20 strain used in this study has a high level of SpeB production (Chiang-Ni et al., 2009; Barnett et al., 2013). Our results showed that inactivation of SLO production in the capsule-deficient animal-passage *covS* mutant, but not inactivation of the SpeB protease activity in the capsule-deficient A20 strain, attenuated bacterial cytotoxicity on PMA-activated U937 cells (Figures 3D,E). These results suggest that the SpeB does not have critical roles in GAS survival in the intracellular niche of phagocytic cells. Furthermore, these results also provide an insight to explain why the SpeB-negative *covS* mutant can be selected by the immune system during infection.

GAS is one of the most important pathogens causing severe soft-tissue infections such as cellulitis and necrotizing fasciitis (Olsen and Musser, 2010). The presentations of cellulitis and necrotizing fasciitis at the early stage are similar. Nonetheless, necrotizing fasciitis is a rapidly progressive disease with severe tissue destruction and high mortality (Stevens and Bryant, 2017). Therefore, the early diagnosis and treatment of necrotizing fasciitis are crucial (Olsen and Musser, 2010; Stevens and Bryant, 2017). GAS strains with mutations in the *covR* or *covS* genes are more frequently isolated from patients with necrotizing fasciitis than from patients with mild pharyngitis (Ikebe et al., 2010; Friaes et al., 2015b). Eliminating these invasive mutants in the early stage of infection may prevent severe soft-tissue destructions. The present study showed that the treatment of infected phagocytic cells with clindamycin, but not penicillin, attenuated the virulence of intracellular capsule-deficient animal-passage *covS* mutant (Figure 5). These results not only support that clindamycin should be used to treat patients with invasive GAS infection, but also suggest that killing or inactivating intracellular GAS could be a potential strategy to prevent severe tissue destructions of invasive GAS infection.

Although considered an extracellular pathogen, GAS is reported to survive and replicate in phagocytic cells and several other types of mammalian cells. In addition, studies have suggested that intracellular GAS has important roles in chronic infection, recurrent infection, asymptomatic infection, and failure of penicillin treatment (Osterlund et al., 1997; Sela and Barzilai, 1999; Podbielski et al., 2003; Kaplan et al., 2006; Oliver et al., 2007). The present study further suggests that intracellular invasive *covS* mutants can have important roles in the development of severe invasive GAS infection.

## Author Contributions

CC-N, C-HL, and C-HC conceived or designed the study. Y-AS acquired, analyzed, or interpreted the data. CC-N wrote the manuscript.

## Conflict of Interest Statement

The authors declare that the research was conducted in the absence of any commercial or financial relationships that could be construed as a potential conflict of interest.
