# Supplementary material for: Cytotoxicity and Survival Fitness of Invasive covS Mutant of Group A Streptococcus in Phagocytic Cells
Source: Front Microbiol. 2018 Oct 30;9:2592. doi: 10.3389/fmicb.2018.02592 (PMC6218877; doi:10.3389/fmicb.2018.02592)
Supplement: Supplementary file 1 [file Data_Sheet_1.docx]

**
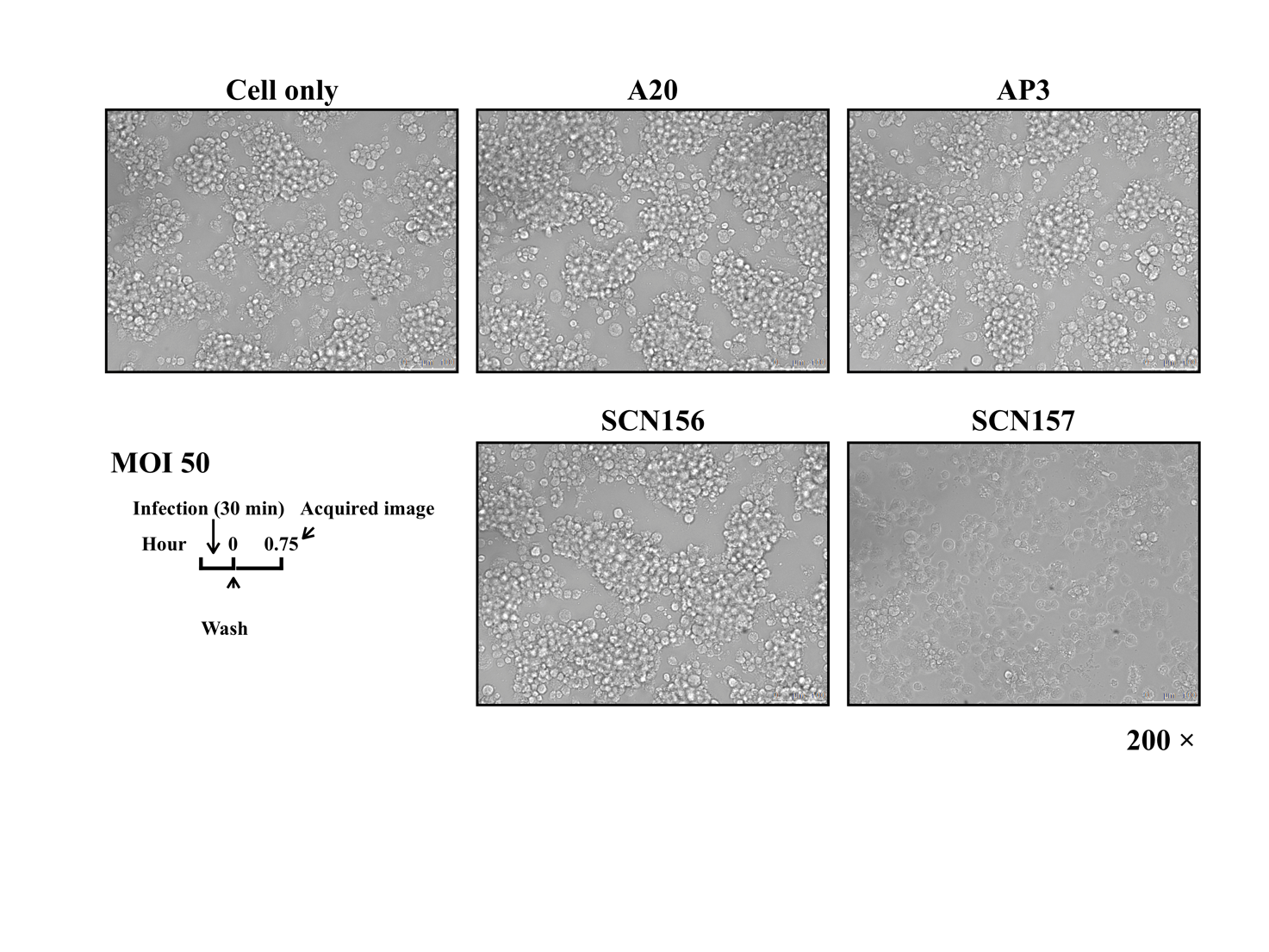
**

**SUPPLEMENTARY FIGURE 1 Morphology of the wild-type A20 strain, animal-passage *covS* mutant AP3, and their capsule-deficient strains (SCN156 and SCN157, respectively) infected U937 cells.** PMA-activated U937 cells were infected by bacteria (MOI = 50) for 30 min. After infection, infected cells were washed and incubated for another 45 min. Images of infected cells were directly acquired by a phase-contrast microscope without staining. The lower-left panel shows the infection protocol.

**
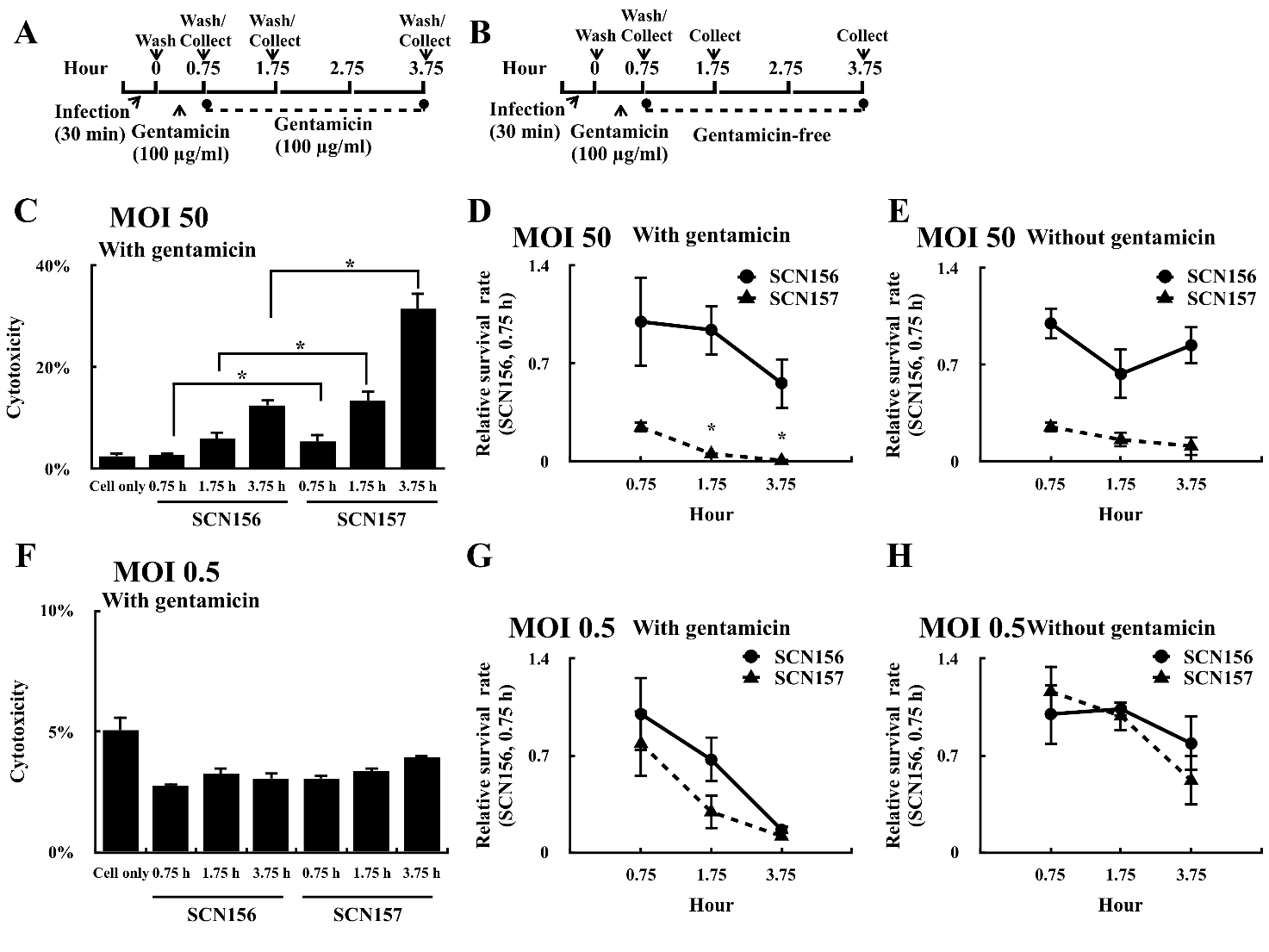
**

**SUPPLEMENTARY FIGURE 2 Cytotoxicity and activity of resistance to PMA-activated U937 cell killing of capsule-deficient A20 (SCN156) and AP3 (SCN157) in the presence or absence of gentamicin treatment after infection.** (A) and (B) Infection protocols for gentamicin-containing and gentamicin-free culture conditions. (C) and (F) Cytotoxicity of SCN156 and SCN157 on PMA-activated U937 cells at MOI of 50 or 0.5. (D) (E) (G) and (H) The survival rate of SCN156 and SCN157 in the presence or absence of gentamicin treatment after infection (MOI = 50 or 0.5). In gentamicin-containing culture, the infected cells were washed, and the number of intracellular bacteria was determined. In gentamicin-free culture, infected cells and culture media were collected for determining the number of survived bacteria. Error bars represent the standard deviations. *, *p* < 0.05


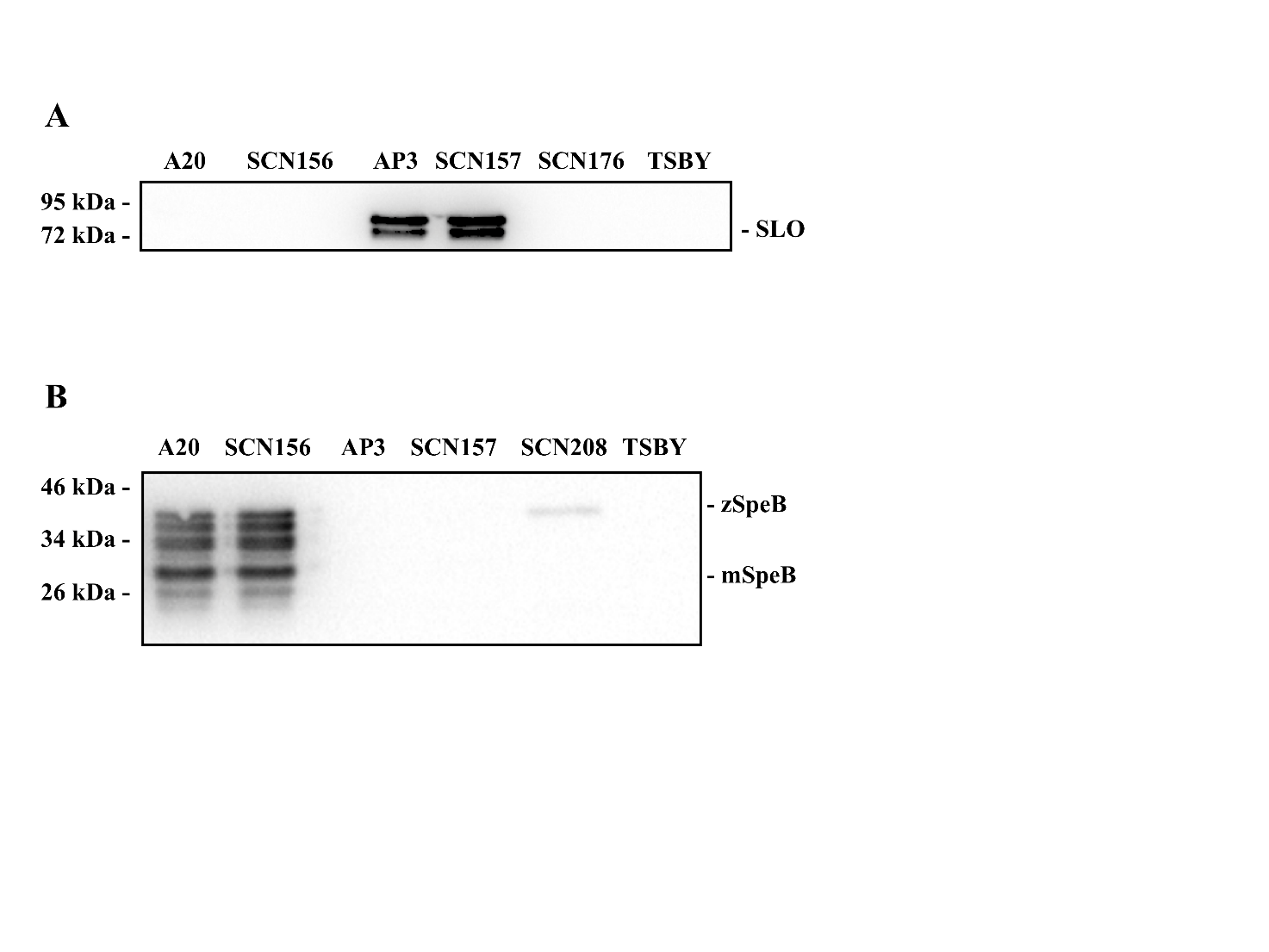


**SUPPLEMENTARY FIGURE 3 The SLO and SpeB expression in the wild-type A20 strain, animal-passage *covS* mutant AP3, their capsule-deficient mutants (SCN156 and SCN157, respectively), *slo* mutant of SCN157 (SCN176), and SpeB protease-inactivated mutant of SCN156 (SCN208).** Bacteria were cultured in TSBY broth to the stationary phase of growth. The 30 µL of culture supernatants were collected and analyzed with western blot. (A) The SLO expression in A20, AP3, their capsule-deficient mutants, and *slo* mutant of capsule-deficient AP3 strain (SCN176). (B) The expression of zymogen- and mature-form of SpeB proteins in A20, AP3, their capsule-deficient mutants, and SpeB protease-inactivated mutant of capsule-deficient A20 strain (SCN208). SpeB is secreted as the 48 kDa zymogen-form protein (zSpeB) and processing into the 28 kDa mature-form protein (mSpeB). TSBY is the culture broth and served as the negative control.
